# Multiple physical and mental health comorbidity in adults with intellectual disabilities: population-based cross-sectional analysis

**DOI:** 10.1186/s12875-015-0329-3

**Published:** 2015-08-27

**Authors:** Sally-Ann Cooper, Gary McLean, Bruce Guthrie, Alex McConnachie, Stewart Mercer, Frank Sullivan, Jill Morrison

**Affiliations:** Mental Health and Wellbeing group, Institute of Health and Wellbeing, University of Glasgow, Administrative Building, Gartnavel Royal Hospital, 1055 Great Western Road, Glasgow, G12 0XH UK; General Practice and Primary Care group, Institute of Health and Wellbeing, University of Glasgow, 1, Horselethill Road, Glasgow, G12 9LX, UK; Quality, Safety and Informatics Research Group, Population Health Sciences Division, University of Dundee, Mackenzie Building Kirsty Semple Way, Dundee, DD2 4BF UK; Robertson Centre for Biostatistics, Institute of Health and Wellbeing, University of Glasgow, Level 11, Boyd Orr Building, University Avenue, Glasgow, G12 8QQ UK; UTOPIAN FMTU, North York General Hospital, Department of Family & Community Medicine and Dalla Lana School of Public Health, University of Toronto, Toronto, ON M2K 1E1 Canada

**Keywords:** Intellectual disabilities, Mental retardation, Multi-morbidity, Physical health, Mental health, Inequalities, Deprivation

## Abstract

**Background:**

Adults with intellectual disabilities have increased early mortality compared with the general population. However, their extent of multimorbidity (two or more additional conditions) compared with the general population is unknown, particularly with regards to physical ill-health, as are associations between comorbidities, neighbourhood deprivation, and age.

**Methods:**

We analysed primary health-care data on 1,424,378 adults registered with 314 representative Scottish practices. Data on intellectual disabilities, 32 physical, and six mental health conditions were extracted. We generated standardised prevalence rates by age-groups, gender, and neighbourhood deprivation, then calculated odds ratio (OR) and 95 % confidence intervals (95 % CI) for adults with intellectual disabilities compared to those without, for the prevalence, and number of condition.

**Results:**

Eight thousand fourteen (0.56 %) had intellectual disabilities, of whom only 31.8 % had no other conditions compared to 51.6 % without intellectual disabilities (OR 0.26, 95 % 0.25–0.27). The intellectual disabilities group were significantly more likely to have more conditions, with the biggest difference found for three conditions (10.9 % versus 6.8 %; OR 2.28, 95 % CI 2.10–2.46). Fourteen physical conditions were significantly more prevalent, and four cardiovascular conditions occurred less frequently, as did any cancers, and chronic obstructive pulmonary diseases. Five of the six mental health conditions were significantly more prevalent. For the adults with intellectual disabilities, no gradient was seen in extent of multimorbidity with increasing neighbourhood deprivation; indeed findings were similar in the most affluent and most deprived areas. Co-morbidity increased with age but is highly prevalent at all ages, being similar at age 20–25 to 50–54 year olds in the general population.

**Conclusions:**

Multi-morbidity burden is greater, occurs at much earlier age, and the profile of health conditions differs, for adults with intellectual disabilities compared with the general population. There is no association with neighbourhood deprivation; people with intellectual disabilities need focussed services irrespective of where they live, and at a much earlier age than the general population. They require specific initiatives to reduce inequalities.

**Electronic supplementary material:**

The online version of this article (doi:10.1186/s12875-015-0329-3) contains supplementary material, which is available to authorized users.

## Background

Adults with intellectual disabilities are thought to experience health inequalities and earlier age of death compared with the general population [1]. However, there is little reported information on their wider experience of multimorbidity/comorbidity (two or more conditions additional to the intellectual disabilities) in this population across the adult lifespan. Comorbidity is clinically important, as it may require a different management approach to the care of an individual disease, and may introduce pharmacological contraindications. There is increasing awareness of its clinical importance, due to the relatively recent studies of multi-morbidity in the general population showing that it starts to become more common over the age of 50 and increases in the elderly [2]. In people with intellectual disabilities, rates of individual disorders have been previously reported, for example, a point-prevalence of 40 % for additional mental ill-health [3], 30 % for epilepsy [4], and 50 % for gastro-oesophageal reflux disorder [5]. This might suggest that multi-morbidity would be a particular problem for this population, but we have only been able to find two previous studies on the topic, both of which were focussed only on older people with intellectual disabilities [6, 7]. Both reported high rates of multimorbidity/comorbidity; 71 % in 695 older persons with intellectual disabilities [6], and 80 % in 1047 older persons receiving paid support [7]. These studies did not drawn direct comparisons with rates in the general population living in the same areas, nor at the same age.

The extent of multimorbidity is higher in the general population living in more deprived neighbourhoods [2]. It is therefore important to examine if this is also true for people with intellectual disabilities, since this would indicate higher needs in this population which may need specific organisation to meet. Both children and adults with intellectual disabilities are more likely to live in more deprived areas [8–11]. However, the impact this has on their health and health care has been little studied [10].

This study was undertaken to quantify the extent of recorded ill-health and comorbidity experienced by adults with intellectual disabilities compared with the general population, and to measure the associations between neighbourhood deprivation, age, and comorbidity in adults with intellectual disabilities.

## Methods

We used data from the Primary Care Clinical Informatics Unit at the University of Aberdeen for all 1,424,378 registered patients aged 18 and over, who were alive and permanently registered with one of 314 Scottish general practices on March 31, 2007 [12]. The dataset is representative of the whole Scottish population in terms of age, sex, and socioeconomic deprivation, with a more detailed explanation available elsewhere [2].

Data on the presence of intellectual disabilities, 32 common chronic physical health conditions and six mental health conditions were extracted (definitions are provided in Additional file 1: Appendix 1). We defined intellectual disabilities using a set of Read Codes based on definitions used by NHS Scotland Information Services and from the Quality & Outcomes Framework (Additional file 1: Appendix 2).

Neighbourhood deprivation was measured using the Carstairs deprivation score divided into quintiles (from most affluent to most deprived) [13]. The Carstairs score is based on postcode of residence and is widely used in healthcare research as a measure of socioeconomic status.

To control for differences between the two populations in age, gender and deprivation levels we adopted a similar approach to that undertaken in previous papers [14, 15] and generated standardised prevalence rates by age groups (18 to 24 years; 25 to 34; 35 to 44; 45 to 54; 55 to 64; 65 to 74 and 75 and over), gender, and deprivation quintile using the direct method. These age-gender-deprivation standardised rates were then used to calculate odds ratio (ORs) and 95 % confidence intervals (95 % CI) for the adults with intellectual disabilities compared to those without (controls), for the prevalence of 32 physical conditions and six mental health conditions, as well as by the number of overall conditions and the number of physical and mental health conditions.

We report by age group and gender, differences between those with and without intellectual disabilities in the percentage of individuals with two or more physical conditions and two or more mental health conditions. We used t tests to analyse differences between groups and one-way analysis of variance for differences across age groups and deprivation quintiles. For all statistical analyses, a *p*-value less than 0 · 05 was considered statistically significant. All analyses were performed in Stata version 13.

We also compared the extent of monitoring of blood pressure in the over 50 years with and without intellectual disabilities to see if there were any monitoring/recording differences. Blood pressure measurement is routinely conducted in practices at this age.

The NHS Grampian Research Ethics Service approved the anonymous use of these data for research purposes.

## Results

### Demographics

There were 8014 (0.56 % of the sample) patients with a Read Code for intellectual disabilities recorded (Table 1). This is similar to previously reported prevalence rates from another area of Scotland where there was rigorous checking of the population [2]. Men were over-represented in the intellectual disabilities group compared to controls (56.4 vs. 49.1 % for controls; *p* < 0.001). Individuals with recorded intellectual disabilities were on average younger (mean age 43.1 vs. 48.0 years for controls; *p* < 0.001), with only 11.4 % aged 65 or over compared to 20.6 % of controls. The adults with intellectual disabilities were also more likely to live in areas of high social deprivation, with just over a quarter (25.3 %) with intellectual disabilities resident in the most deprived quintile of postcodes compared to 17.8 % of controls (*p* < 0.001).

**Table 1:** Age, gender, and deprivation status, intellectual disabilities versus controls

Variable	Intellectual disabilities	No intellectual disabilities
	Number (%)	Number (%)
Total (%)	8014 (0.6 %)	1,416,364 (99.4 %)
Gender (% male)	4518 (56.4 %)	694,911 (49.1 %)
Mean Age (sd)	43.1 (15.8)	48.0 (18.3)
Age group		
18–24	1192 (14.9)	150,501 (10.6)
25–34	1419 (17.7)	227,977 (16.1)
35–44	1811 (22.6)	277,182 (19.6)
45–54	1639 (20.5)	252,155 (17.8)
55–64	1116 (13.9)	218,217 (15.4)
65–74	593 (7.4)	154,687 (10.9)
75 and above	244 (3.0)	135,645 (9.6)
Deprivation Quintile		
Least Deprived	959 (11.9)	271,070 (19.1)
2	1421 (17.7)	302,733 (21.4)
3	1849 (23.0)	320,398 (22.6)
4	1757 (21.9)	269,627 (19.0)
Most Deprived	2028 (25.3)	252,536 (17.8)

All difference significant at *p* < 0.001

### Comorbidities

Overall, 31.8 % of individuals with intellectual disabilities had no other conditions compared to 51.6 % of controls with no recorded condition (Table 2). The intellectual disabilities group were significantly more likely to have more of all the specified number of conditions after standardising for age, sex and social deprivation, with the smallest difference found for one condition (intellectual disabilities 27.5 % vs. controls 21.3 %; OR 1.48, 95 % CI 1.41–1.55), and the biggest difference found for three conditions (intellectual disabilities 10.9 % vs. controls 6.8; OR 2.28, 95 % (CI 2.10–2.46) (Table 2).

**Table 2:** Prevalence and odds ratio for number and type of comorbidities (standardised by age, gender, and deprivation score)

	Intellectual disabilities	No Intellectual disabilities	Odds ratio (95 % CI) (standardised by age, gender and deprivation)
	N (prevalence %)	N (prevalence %)	
	*N* = 8014 (0.6 %)	*N* = 1,416,364 (99.4 %)	
Total number of morbidities^a^			
None	2552 (31.8)	731,181 (51.6)	0.26 (0.25–0.27)
One	2207 (27.5)	301,743 (21.3)	1.48 (1.41–1.55)
Two	1498 (18.7)	162,371 (11.5)	2.13 (1.99–2.26)
Three	874 (10.9)	96,256 (6.8)	2.28 (2.10–2.46)
Four	471 (5.9)	57,231 (4.0)	2.07 (1.87–2.29)
Five or more	412 (5.1)	67,582 (4.8)	1.60 (1.44–1.79)
Total number of physical conditions			
None	3087 (38.5)	799,884 (56.5)	0.27 (0.25–0.29)
One	2397 (29.9)	294,613 (20.8)	1.79 (1.71–1.80)
Two	1428 (17.8)	149,477 (10.5)	2.50 (2.34–2.66)
Three	631 (7.9)	83,016 (5.9)	1.96 (1.79–2.15)
Four	309 (3.9)	45,587 (3.3)	1.76 (1.56–1.99)
Five or more	162 (2.0)	43,787 (3.1)	0.95 (0.81–1.12) *p* = 0.59
Total number of mental health conditions^a^			
None	5878 (73.4)	1,205,242 (85.1)	0.42 (0.40–0.44)
One	1577 (19.7)	160,958 (11.4)	2.10 (1.99–2.23)
Two	471 (5.9)	43,232 (3.1)	2.26 (2.05–2.48)
Three or more	88 (1.1)	6932 (0.5)	2.43 (1.96–3.00)

One-way analysis of variance

All difference significant at *p* < 0.001 except where marked

^a^Excluding intellectual disabilities

When restricting analysis only to physical health comorbidities the adults with intellectual disabilities were far less likely to have no physical conditions (intellectual disabilities 38.5 % vs. controls 56.5 %; OR 0.27, 95 % CI 0.25–0.29) and more likely to have one to four physical conditions, with the biggest difference found for two physical conditions (intellectual disabilities 17.8 % vs. controls 10.5 %; OR 2.50, 95 % CI 2.34–2.66), but there were no differences found for five or more conditions.

People with intellectual disabilities were less likely to have no recorded mental health condition compared to controls (intellectual disabilities 73.4 % vs. controls 85.1 %; OR 0.42, 95 % CI 0.40–0.44) and twice as likely to have one, two and three or more mental health conditions, than people with no intellectual disabilities.

### Physical health individual conditions

For the intellectual disabilities group, 14 out of 32 physical conditions were significantly more prevalent relative to controls, 11 were significantly less prevalent, with 7 conditions showing no significant differences (Table 3). The largest differences, after standardisation for age, sex and deprivation, were for epilepsy (OR 31.03, 95 % CI 29.23–32.92) constipation (OR 11.19, 95 % CI 10.97–12.68) and visual impairment (OR 7.81, 95 % CI 6.86–8.89). Five further conditions were more than twice as likely to be prevalent in those with intellectual disabilities compared to controls (hearing loss, eczema, dyspepsia, thyroid disorders and Parkinson’s Disease or Parkinsonism). Of the eleven conditions for which the relative prevalence for the adults with intellectual disabilities was lower, four were cardiovascular related (coronary heart disease OR 0.43, 95 % CI 0.37–0.51, peripheral vascular disease OR 0.44, 95 % CI 0.33–0.60, hypertension OR 0.72, 95 % CI 0.66–0.78 and atrial fibrillation OR 0.83, 95 % CI 0.61–0.98). Lower prevalence in those with intellectual disabilities also included any cancer over the last 5 years (OR 0.69, 95 % CI 0.58–0.83) and chronic obstructive pulmonary diseases (OR 0.84, 95 % CI 0.73–0.97).

**Table 3:** Actual prevalence rates, and standardised odds ratios for individual physical conditions. Conditions are ordered by size of standardised odds ratio (largest to smallest)

Condition	Intellectual disabilities	No Intellectual disabilities	Odds ratio (95 % CI) (standardised by age, gender and deprivation score)
	Number (%)	Number (%)	
Epilepsy	1508 (18.8)	10,876 (0.8)	31.03 (29.23–32.92)
Constipation	1118 (14.0)	35,298 (2.5)	11.19 (10.97–12.68)
Visual impairment	258 (3.2)	8120 (0.6)	7.81 (6.86–8.89)
Parkinson’s disease and Parkinsonism	28 (0.4)	2713 (0.2)	2.83 (1.95–4.13)
Hearing loss	657 (8.2)	54,077 (3.8)	2.81 (2.59–3.06)
Dyspepsia	822 (10.3)	78,382 (5.5)	2.46 (2.28–2.65)
Psoriasis or eczema	132 (1.7)	10,237 (0.7)	2.42 (2.03–2.87)
Thyrotoxicosis/thyroid disorders inc hypothyroidism	629 (7.9)	71,314 (5.0)	2.36 (2.173–2.58)
Bronchiectasis	20 (0.3)	2794 (0.2)	1.68 (1.08–2.61) *p* = 0.02
Diabetes	531 (6.6)	74,300 (5.3)	1.63 (1.49–1.79)
Migraine	59 (0.7)	9192 (0.7)	1.32 (1.02–1.71) *p* = 0.03
Active asthma	575 (7.2)	83,930 (5.9)	1.26 (1.16–1.38)
Painful condition	695 (8.7)	125,436 (8.9)	1.20 (1.10–1.30)
Stroke or transient ischaemic attack	171 (2.1)	36,374 (2.6)	1.19 (1.02–1.37) *p* = 0.02
Glaucoma	72 (0.9)	15,847 (1.1)	1.17 (0.92–1.48) *p* = 0.18
Chronic kidney disease	135 (1.7)	33,431 (2.4)	1.11 (0.93–1.32) *p* = 0.22
Heart failure	82 (1.0)	18,817 (1.3)	1.11 (0.89–1.43) *p* = 0.33
Irritable bowel syndrome	248 (3.1)	51,889 (5.7)	0.97 (0.86–1.11) *p* = 0.74
Chronic obstructive pulmonary diseases (COPD)	209 (2.6)	52,898 (3.7)	0.84 (0.73–0.97)
Atrial fibrillation	71 (0.9)	23,905 (1.7)	0.83 (0.61–0.98) *p* = 0.03
Viral hepatitis	7 (0.1)	1168 (0.1)	0.82 (0.39–1.74) *p* = 0.62
Inflammatory bowel disease	40 (0.5)	9711 (0.7)	0.82 (0.60–1.13) *p* = 0.23
Hypertension	774 (9.7)	233,540 (16.5)	0.72 (0.66–0.78)
Any new cancer in the last 5 years	131 (1.6)	43,533 (3.1)	0.69 (0.58–0.83)
Prostate disease	41 (0.5)	15,192 (1.1)	0.60 (0.44–0.82) *p* = 0.01
Inflammatory arthritis and related conditions inc gout	151 (2.2)	57,857 (4.1)	0.57 (0.48–0.67)
Cirrhosis/chronic liver disease/alcoholic liver disease	7 (0.1)	2605 (0.2)	0.49 (0.23–1.04) *p* = 0.06
Multiple sclerosis	9 (0.1)	3838 (0.3)	0.49 (0.25–0.96) *p* = 0.03
Diverticular disease	66 (0.8)	33,747 (2.4)	0.49 (0.39–0.63)
Peripheral vascular disease (PVD)	46 (0.7)	23,194 (1.6)	0.44 (0.33–0.60)
Chronic sinusitis	24 (0.3)	9141 (0.6)	0.44 (0.26–0.62)
Coronary heart disease	160 (2.0)	81,307 (5.7)	0.43 (0.37–0.51)

One way analysis of variance

All differences significant at *p* < 0.001 except where stated

### Mental health conditions

Table 4 highlights that the adults with intellectual disabilities had significantly higher prevalence for five of the mental health conditions with no significant difference found for anorexia/bulimia. The biggest difference after standardisation for age, sex and deprivation was for schizophrenia/bipolar (OR 7.16, 95 % CI 6.49–7.89), followed by anxiety (OR 2.62, 95 % CI 2.41–2.84). The highest prevalence for a mental health condition was found for depression with prevalence 15.8 % for those with intellectual disabilities compared to 10.1 % of controls (OR 1.88, 95 % CI 1.76–2.00). (The lower prevalence of dementia in the raw data is because the proportion of people with intellectual disabilities who have dementia is small, given the age distribution of people with intellectual disabilities. However the OR is standardized for age, and so reflects the fact that people with intellectual disabilities, particularly Down syndrome, experience dementia at a much earlier age than the general population.)

**Table 4:** Prevalence and odds ratios for individual mental health conditions (standardised by age, gender, and deprivation score). Conditions are ordered by size of odds ratio (largest to smallest)

Condition	Intellectual disabilities	No Intellectual disabilities	Odds ratio (95 % CI) (standardised by age, gender and deprivation)
	total number (prevalence)	total number (prevalence)	
Schizophrenia (and related non-organic psychosis) or bipolar disorder	448 (5.6)	12,045 (0.9)	7.16 (6.49–7.89)
Anxiety & other neurotic, stress related & somatoform disorders	649 (8.1)	55,077 (3.9)	2.62 (2.41–2.84)
Dementia	84 (0.8)	11,612 (1.1)	2.22 (1.78–2.77)
Depression	1267 (15.8)	142,676 (10.1)	1.88 (1.76–2.00)
Anorexia or bulimia	37 (0.5)	5269 (0.4)	1.31 (0.95–1.82) *p* = 0.09
Alcohol misuse	304 (3.8)	42,060 (3.0)	1.18 (1.05–1.33)

One way analysis of variance

All difference significant at *p* < 0.001 except where stated

### Effect of deprivation

**Fig. 1:**
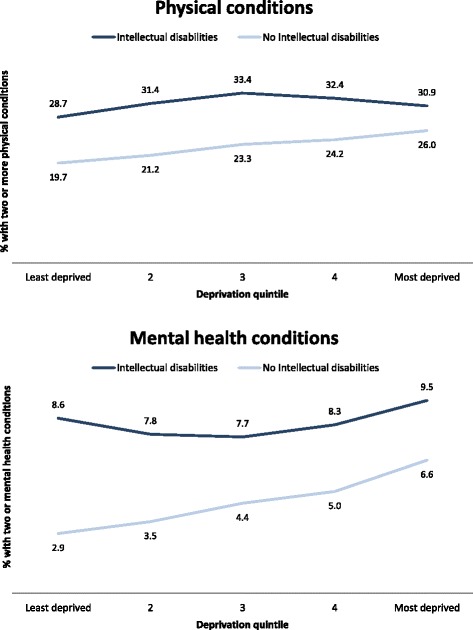
Proportion of people with two or more physical conditions, and two or more mental health conditions (excluding intellectual disabilities) by deprivation quintile (standardised by age and sex)

Figure 1 shows the percentage of individuals with two or more physical conditions and two or more mental health conditions by deprivation quintile after age and sex standardisation. Prevalence is higher in the intellectual disabilities group for both physical and mental health conditions across all quintiles. A clear gradient is seen for the general population in whom the percentage with two or more conditions increases as the extent of neighbourhood deprivation increases. No such gradient is seen for the adults with intellectual disabilities; indeed the proportion with two or more physical health conditions, or two or more mental health conditions, is similar in both the most affluent and most deprived areas.

### Effect of age and gender

**Fig. 2:**
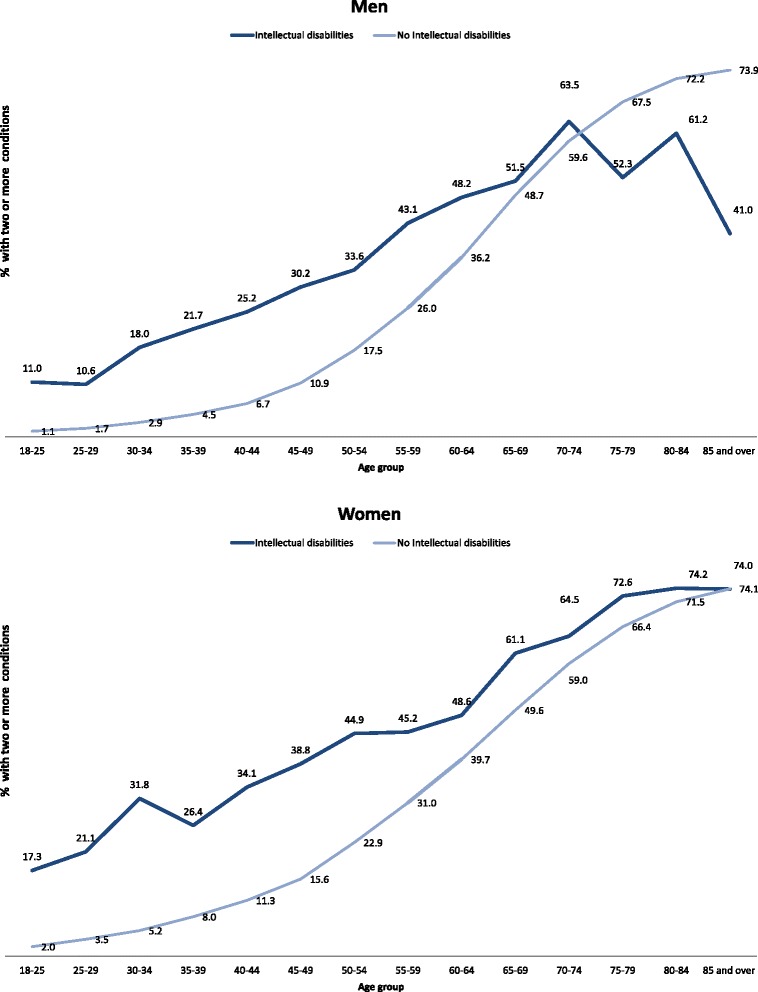
Proportion of people with two or more physical conditions by gender and age group (standardised by deprivation)

Figure 2 shows the percentage of individuals with two or more physical conditions by age group and gender after standardisation by deprivation. Prevalence is higher in the intellectual disabilities group for both men and women for all age groups with the exception of those aged 75 and above for males. Differences peak at 45–49 for males and 50–54 for females. Women have higher rates for both groups across all ages. In the adults with intellectual disabilities, co-morbidity increased with age but is highly prevalent at all ages, with its extent at age 20–25 being similar to that of 50–54 year olds in the general population.

**Fig. 3:**
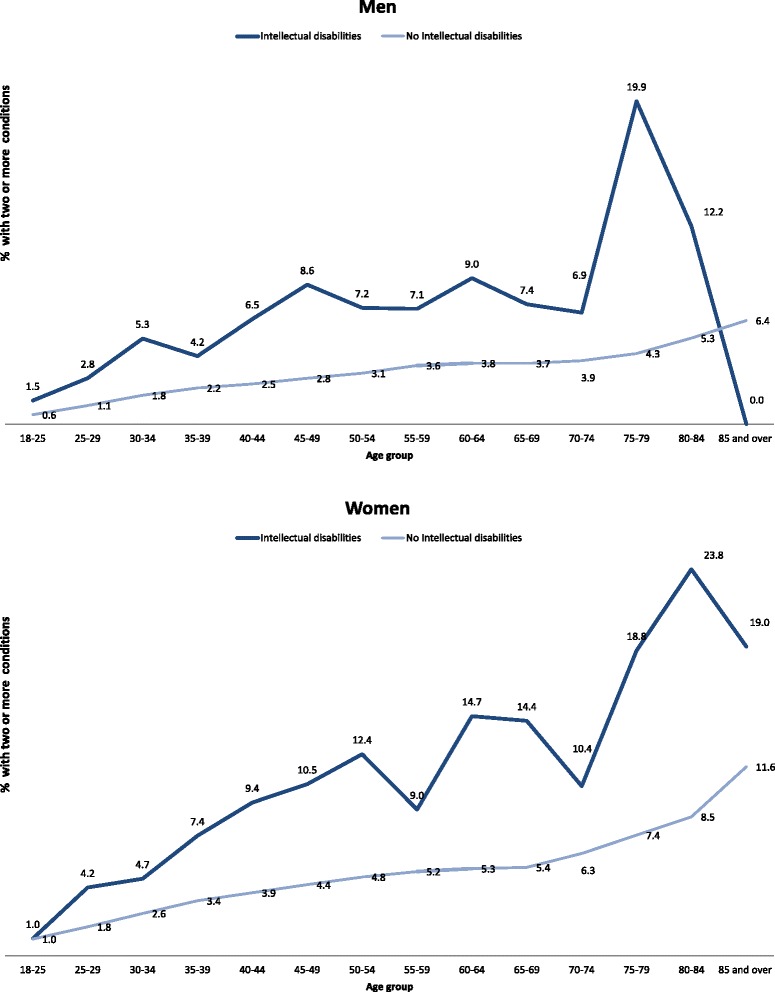
Proportion of people with two or more mental health conditions by gender and age group (standardised by deprivation)

Figure 3 shows the percentage of individuals with two or more mental health conditions by age group and gender, after standardisation by deprivation. A similar trend was found as with physical conditions, with prevalence consistently higher in the intellectual disabilities group for both men and women, and higher rates in women for both groups across all ages.

### Monitoring/recording

There was no difference in blood pressure monitoring in our population; 83.3 % of the people with intellectual disabilities aged 50 and over had a blood pressure recorded in the previous three years compared to 84.9 % of those without intellectual disabilities.

## Discussion

### Key results

The extent of multi-morbidity experienced by adults with intellectual disabilities, and its direct comparison with the general population, is a novel and important finding, with implications for services, including the age at which they are likely to be needed. Consequently, any policy initiatives or guidelines on multi-morbidity need to be relevant at a much earlier age in people with intellectual disabilities. This difference has not been previously reported. Morbidity burden and multimorbidity is higher in the population with intellectual disabilities than in the general population, due to higher rates of some physical conditions, for example epilepsy, gastro-intestinal disorders, and sensory impairments, and higher rates of a range of mental health conditions. Whereas multi-morbidity is common in the general population only in older age groups, particularly those aged 50–54 and older, it is common in all age groups in adults with intellectual disabilities. The pattern of disease also differs from the general population with some conditions being less common, such as cardiovascular disease. Additionally, prevalence of multi-morbidity did not follow the typical gradient seen in the general population across areas of increasing neighbourhood deprivation, importantly highlighting that services are equally needed in all areas.

Two previous studies from the Netherlands and Ireland have reported high rates of multimorbidity in older people with intellectual disabilities [6, 7], and we also found this, and extended this finding down the age range to all adults with intellectual disabilities.

### Strengths and limitations

Scottish GP practices have held a register of people with intellectual disabilities since a change in their contract introducing pay-for-performance, which precedes the data extraction this study used. Intellectual disability is a lifetime diagnosis, and once coded at birth or in childhood this remains on the medical record indefinitely. The sample with intellectual disabilities appears to be representative, and benefits from its very large size. As expected, compared with people without intellectual disabilities, there were more men, they were younger, and they were more likely to live in deprived areas. Rates of morbidity were compared with the general population registered at the same general practices, and standardised by age, gender, and neighbourhood deprivation. It is possible that some people with intellectual disabilities were not coded as such, for example people with Down syndrome, however the prevalence of the population identified is similar to that reported for adults with intellectual disabilities in a recent meta-analysis of prevalence studies (0.5 %) [16], and the odds ratio for dementia for the intellectual disabilities group compared with the general population suggests people with Down syndrome, who have dementia at a much earlier age than the general public, were included.

There may be under-reporting of health conditions in the population with intellectual disabilities. This may be so for conditions that are not overtly obvious to paid carers, or where carers attribute the effects of conditions to other reasons. The similarity in extent of blood pressure recording in the population with intellectual disabilities compared with the general population is reassuring in this regard. If there was under-reporting, then the difference between the two groups would be even more marked than that we report, and the key message of our paper still stands i.e. that multi-morbidity is markedly more common in adults with intellectual disabilities than in the general population, and occurs at a much younger age.

Problem behaviours, which occur in 22.5 % of adults with intellectual disabilities [17] were not included in the study, due to the lack of suitable Read codes for these disorders, hampering their recording/consistent recording. Comparable problem behaviours are rare in the general population, hence the extent of the difference in multimorbidity would have been greater if these could have been included. We also did not include autism and attention deficit hyperactivity disorder, both of which are known to be more common in people with intellectual disabilities than in the general population. Conditions are coded during routine health care, including primary care encounters and based on letters from secondary care, and there could be some variation between practices.

We do not have information on type of accommodation/support the people with intellectual disabilities had.

### Interpretation of findings

Some causes of intellectual disabilities also cause physical and/or mental ill-health, for example Down syndrome is associated with thyroid disorder and sensory impairments; however, Down syndrome accounts for only about 15 % of the population with intellectual disabilities. Adults with intellectual disabilities are also more likely to lead sedentary lives and not exercise [18], have more mobility problems [19], obesity [20], and are less likely to eat healthily [21] than the general public, and about a quarter take antipsychotic drugs [22], which may contribute to some of these conditions. They are also more likely to be prescribed multiple drugs, which can adversely affect health through side-effects and drug interactions [21]. They do not always have the knowledge or understanding to make healthy choices, and are reliant on others for support and communication. These issues are often compounded by difficulties accessing the health services they need.

Eleven of the conditions were recorded statistically less commonly in adults with intellectual disabilities than in the general population. The lower rates of smoking and alcohol use among the population with intellectual disability may well account for several of these conditions being diagnosed at a lower frequency, particularly cardiovascular disease and chronic obstructive pulmonary disease. The majority of adults with intellectual disabilities do not drink alcohol at all, although some do misuse it, and at a slightly higher rate than the general population in this study.

Despite the higher prevalence of comorbidity experienced by the adults with intellectual disabilities, the extent of their morbidities may be under-recorded. Mental and physical health conditions may be unrecognised, under-investigated and untreated [23–26], with ill-heath presenting late, at more severe stages of disease progression which may be less responsive to treatment. Chronic disease monitoring is also less well addressed [27, 28]. Several factors are implicated, such as limited verbal communication skills, impaired mobility, and problem behaviours. People with intellectual disabilities are reliant on carers recognising they may have a problem and seeking help, and dependent upon carers communicating effectively within the team, and indeed across care teams (e.g. day care team and home care team). Sometimes, health conditions are misattributed by paid carers or health professionals as being part of the adult’s intellectual disabilities (diagnostic overshadowing), and not addressed for this reason. These problems are compounded across the entire lifecourse, rather than just being due to communication problems in late life.

The apparent drop-off in the rate of multimorbidity in men aged 75 and older is likely to be a reflection of the very small numbers in these age groups. Most people with intellectual disabilities do not live to such old ages [29, 30], so these individuals are the “healthy survivors”. Older people with intellectual disabilities typically have milder intellectual disabilities than those who die earlier, and people with milder intellectual disabilities are likely to have fewer health problems than people with more severe intellectual disabilities. Of the total of 4518 men with intellectual disabilities in the study, there were only 60 (1.32 %) aged 75–79, 32 (0.71 %) aged 80–84, and 16 (0.35 %) aged 85 or older. This compares with 24,831 (3.57 %) aged 75–79 out of the total of 694,911 men without intellectual disabilities, 15,921 (2.29 %) aged 80–84, and 11,017 (1.59 %) aged 85 or older.

The lack of association between neighbourhood deprivation and multimorbidity in this population is likely to be due to area based measures of deprivation not accurately reflecting the relative degree of affluence or poverty experienced by people with intellectual disabilities, in the face of the extensive difficulties they have to cope with in life. Many adults with intellectual disabilities are not integrated within their communities. They do not necessarily have shared values and lifestyles with their local community. Rented accommodation in which adults with intellectual disabilities are placed with individual tenancies, or shared tenancies with other adults with intellectual disabilities, tend to be in less affluent areas. One can speculate that their paid carers are more likely to live in the local area, but the adult may still have regular contact with family, whom they grew up with and who may have different levels of affluence and lifestyles compared to the area their adult child with intellectual disabilities now lives in. The interaction of these factors is likely to be complex. Additionally, some of the more congregate care style of housing is more likely to be in affluent areas where there are larger houses; but large group living can result in less individual time from paid carers who are shared by several adults, and less time for community integration. Very few adults with intellectual disabilities have paid employment, so are likely to be of low socio-economic status, and dependant on state benefits, regardless of the area they live in.

### Generalisability of findings

The broader dataset is representative of the Scottish population in terms of age, sex, and deprivation [12]. Intellectual disabilities was found in 0.56 % of the sample. This is slightly higher than the 0.5 % recorded in GP registers for pay-for-performance, reflecting that we used a somewhat broader set of Read Codes (http://www.isdscotland.org/Health-Topics/General-Practice/Quality-And-Outcomes-Framework/ Accessed 23.12.14.). As expected, there were more men than women with intellectual disabilities (as more boys than girls are born with intellectual disabilities), a smaller proportion at older age groups than in the general population (due to premature death [29, 30]), and more lived in areas of neighbourhood deprivation. This suggests that the sample with intellectual disabilities is representative of the Scottish population, and hence that these findings are generalisable.

## Conclusions

This study is important as it demonstrates, in a very large cohort, the increased burden of multi-morbidity experienced by adults with intellectual disabilities compared with the general population, and with much earlier age of onset. Their extent of co-morbidity at age 20–25 is similar to that of the general population aged 50–54. Additionally, their profile of health conditions differs from the general population and does not have the same associations with neighbourhood deprivation. There may also be under-recording of some conditions due to access difficulties, including carers not recognising problems nor seeking health care, and conditions not being diagnosed or managed appropriately [30]. The implication is that policy initiatives to benefit the majority of the population (i.e. the general population) are unlikely to equally benefit the population with intellectual disabilities, despite their greater overall morbidity. Examples include focussing initiatives and resources in areas of greatest neighbourhood deprivation, and smoking cessation programmes. Assumptions about people with intellectual disabilities’ health profiles and determinants of health cannot necessarily be drawn from the general population. Reducing the health inequality gap will require specific initiatives for adults with intellectual disabilities, and we have demonstrated that people with intellectual disabilities need focussed services irrespective of where they live, and from an early age. This presents challenges for primary care, and highlights a potentially key role for paid carers in supporting access to and across services.

## Additional file

Additional file 1:
**Appendix 1: Data dictionary.** Appendix 2: Read Codes used to define the presence of intellectual disability (DOCX 21 kb)

## Abbreviations

CI: Confidence intervals
NHS: National Health Service
OR: Odds ratio
