# Supplementary material for: Multiple physical and mental health comorbidity in adults with intellectual disabilities: population-based cross-sectional analysis
Source: BMC Fam Pract. 2015 Aug 27;16:110. doi: 10.1186/s12875-015-0329-3 (PMC4551707; doi:10.1186/s12875-015-0329-3)
Supplement: Additional file 1: — Appendix 1: Data dictionary. Appendix 2: Read Codes used to define the presence of intellectual disability (DOCX 21 kb) [file 12875_2015_329_MOESM1_ESM.docx]

### Appendix 1. Data dictionary

**Definitions of the health conditions included in the analyses**

**Morbidities to include**

We specifically sought to include morbidities recommended as core for any multimorbidity measure by a recent systematic review (11 conditions),[^1^](file:///C:\Users\gmclea\Downloads\019%20MultimorbidityPaper_RevisedSupplementaryFileSubmitted.doc#_ENREF_1) diseases included in the Quality and Outcomes Framework (QOF) of the UK General Practice contract (16 conditions at the time the data was recorded),[^2^](file:///C:\Users\gmclea\Downloads\019%20MultimorbidityPaper_RevisedSupplementaryFileSubmitted.doc#_ENREF_2) and long term conditions identified as important for health service planning by NHS Scotland (26 conditions).[^3^](file:///C:\Users\gmclea\Downloads\019%20MultimorbidityPaper_RevisedSupplementaryFileSubmitted.doc#_ENREF_3) The research team, which included four general practitioners, then considered the full list of 78 Read Code Groups used by NHS Scotland to define morbidities leading to general practice consultation (including acute and self-limiting conditions, and administrative problems) and by consensus identified those morbidities which were likely to be chronic (defined as having significant impact over at least the most recent year) and with significant impact on patients in terms of need for chronic treatment, reduced function, reduced quality of life, and risk of future morbidity and mortality.

**Definition of each morbidity**

We used an existing definition of a morbidity wherever possible, using QOF definitions[^2^](file:///C:\Users\gmclea\Downloads\019%20MultimorbidityPaper_RevisedSupplementaryFileSubmitted.doc#_ENREF_2) where available and NHS Scotland Read Code groups[^3^](file:///C:\Users\gmclea\Downloads\019%20MultimorbidityPaper_RevisedSupplementaryFileSubmitted.doc#_ENREF_3) where not. For morbidities which have lifelong implications such as coronary heart disease, we defined the presence of the morbidity on the basis of a relevant Read Code ever being recorded. However, ‘Read Code recorded ever’ is not appropriate for conditions where full prolonged remission or cure is possible. For morbidities where we judged this to be the case, we defined the presence of the morbidity in terms of a Read Code recorded in a defined period (for example, cancer recorded in the previous five years), or in terms of the presence of a Read Code ever and relevant prescribing in the previous year (which is the way in which the UK Quality and Outcomes Framework defines ‘active’ asthma and epilepsy for payment purposes). For some morbidities where initial data exploration identified likely significant under-recording of Read Codes, we defined the presence of the morbidity in terms of a Read Code recorded in the previous year or the receipt of 4 or more relevant prescriptions in the previous year (for example depression), or in some cases solely by receipt of 4 or more prescriptions in the previous year for drugs used specifically for particular morbidities (for example, migraine). A particular issue was the definition of the ‘painful condition’ morbidity. We initially considered including a range of morbidities associated with pain such as osteoarthritis, back pain, and fibromyalgia, but were concerned because of likely under-recording of musculoskeletal problems,[^4^](file:///C:\Users\gmclea\Downloads\019%20MultimorbidityPaper_RevisedSupplementaryFileSubmitted.doc#_ENREF_4) difficulties distinguishing acute or self-limiting episodes from chronic morbidity, and potential double counting since many people with chronic pain acquire a range of diagnostic labels. We therefore chose to define ‘painful condition’ solely on the basis of receipt of four or more specified prescriptions. This is not unproblematic, but we judged that ‘painful condition’ made sense despite its heterogeneity in the same way that ‘depression’ in the primary care population is highly heterogenous, but is a meaningful category in practice. We defined the timing of Read Code recording (ever, or in a defined period) by consensus, based on our clinical knowledge of natural history and general practice morbidity coding practice in the UK. Where prescribing data was used, we chose 4 or more prescriptions in the previous year by consensus as indicating a morbidity with significant impact in the previous year.

**Disease Variables (Read codes based on variable source)**

| **Condition** | **Variable Source** | **Variable Definition** | **Mental/Physical Condition** |
| --- | --- | --- | --- |
| CHD | QOF^2^ | Read code ever recorded | Physical |
| CKD | QOF^2^ | Read code ever recorded | Physical |
| Asthma (active) | QOF^2^ | Read code ever recorded AND any prescription in last year | Physical |
| Atrial fibrillation | QOF^2^ | Read code ever recorded | Physical |
| Epilepsy | QOF^2^ | Read code ever recorded AND epilepsy prescription in last year | Physical |
| Any Cancer last five years | ISD^3^ | Read code first recorded in last 5 years (Group 10-39, 222 & 267) | Physical |
| Thyrotoxicosis/Thyroid disorders (includes hypothyroidism) | ISD^3^ | Read code ever recorded  (Group 44 & 45) | Physical |
| Diabetes | ISD^3^ | Read code ever recorded | Physical |
| Dementia | ISD^3^ | Read code ever recorded  (Group 50) | Mental |
| Alcohol misuse | ISD^3^ | Read code ever recorded  (Group 51) | Mental |
| Intellectual disability | ISD^3^ | Read code ever recorded  (Group 56) | Mental |
| Parkinson’s disease and Parkinsonism | ISD^3^ | Read code ever recorded  (Group 58) | Physical |
| Multiple sclerosis | ISD^3^ | Read code ever recorded  (Group 59) | Physical |
| Stroke or transient ischaemic attack | ISD^3^ | Read code ever recorded  (Group 62 & 84) | Physical |
| Visual impairment | ISD^3^ | Read code ever recorded  (Group 69) | Physical |
| Glaucoma | ISD^3^ | Read code ever recorded  (Group 70) | Physical |
| Hearing loss | ISD^3^ | Read code ever recorded  (Group 73) | Physical |
| Hypertension | ISD^3^ | Read code ever recorded  (Group 75) | Physical |
| Heart failure | ISD^3^ | Read code ever recorded | Physical |
| Peripheral vascular diseases | ISD^3^ | Read code ever recorded  (Group 86) | Physical |
| Chronic sinusitis | ISD^3^ | Read code ever recorded  (Group 98) | Physical |
| Chronic obstructive pulmonary diseases (COPD) | ISD^3^ | Read code ever recorded  (Group 102) | Physical |
| Bronchiectasis | ISD^3^ | Read code ever recorded  (Group 104) | Physical |
| Inflammatory bowel disease | ISD^3^ | Read code ever recorded  (Group 116) | Physical |
| Diverticular disease | ISD^3^ | Read code ever recorded  (Group 118) | Physical |
| Inflammatory arthritis and related conditions inc gout | ISD^3^ | Read code ever recorded  (Group 127) | Physical |
| Prostate disease | ISD^3^ | Read code ever recorded  (Group 140 & 141) | Physical |
| Depression | ISD/definition created by research team | Read code recorded in last year (Group 54)  OR  ≥ 4 anti-depressant prescriptions (excluding low dose tricyclics) in last year | Mental |
| Psoriasis or eczema | definition created by research team | Read code ever recorded (M11% & M12%)  AND  ≥ 4 prescription in last year (BNF 13.4, excluding hydrocortisone, & BNF 13.5) | Physical |
| Viral Hepatitis | definition created by research team | Read code ever recorded  (Codes A7040, A7050, A707., A7070, A7071, A7072, A707X, A70z0 and AyuB1) | Physical |
| Irritable bowel syndrome | definition created by research team | Read code ever recorded (Codes J521%)  OR  ≥ 4 antispasmodic prescription in last year (POM only, exclude kolanticon, alverine citrate & peppermint oil) | Physical |
| Cirrhosis/chronic liver disease/alcoholic liver disease | definition created by research team | Read code ever recorded  (Codes J61%, G8523, G85422 and Jyu71 OR Group 120) | Physical |
| Migraine | definition created by research team | ≥ 4 anti-migraine prescriptions in last year (BNF 040704%, POM only exclude migraleve) | Physical |
| Anxiety & other neurotic, stress related and somatoform disorders | definition created by research team | Read code in last year (Group 55)  OR  ≥ 4 anxiolytic/hypnotic prescriptions  OR  ≥ 4 10/25mg amitriptyline in last year & do not meet the criteria for ‘Pain’ | Mental |
| Dyspepsia | definition created by research team | ≥ 4 prescriptions in last year BNF 0103% excluding antacids  AND NOT  ≥4 NSAIDS OR ≥4 aspirin/clopidogrel | Physical |
| Constipation | definition created by research team | ≥4 prescriptions in last year, BNF 0106% | Physical |
| Pain Condition | definition created by research team | ≥4 specified analgesic prescriptionsin last year (opiods/>8mg co-codamol/NSAIDS)  OR  ≥4 specified anti-epileptics in the absence of an epilepsy Read code in last year(gabapentin, pregabalin and carbamazepine) | Physical |
| Anorexia or bulimia | definition created by research team | Read code ever recorded:  Anorexia (1467., 1612., E271., Eu500, Eu501, R030., R030z)  OR  Bulimia (E2751, Eu502, Eu503, R0360) | Mental |
| Schizophrenia and related non-organic psychosis Or Bipolar disorder | definition created by research team | Read code ever recorded/recorded in last year (mix, see below) Or Lithium prescribed in last 168 days  All of E10% recorded ever  E121. recorded ever  E12z. recorded ever  E13% recorded in last 12 months  E13z. recorded  ever (obviously it’s also in E13% so extends that one for this particular code)  E1z.. recorded ever  E2122 recorded ever  Eu20% recorded ever  Eu22% recorded ever  Eu23% recorded in last 12 months  OR  E110% recorded in last 12 months  E111% recorded ever  E114% recorded ever  E115% recorded ever  E116% recorded ever  E117% recorded ever  E11y. recorded ever  E11y0 recorded ever  E11y1 recorded ever  Eu30% recorded in last 12 months  Eu31 recorded ever  Eu323 recorded ever  Eu333 recorded ever | Mental |

1. Diederichs C, Berger K, Bartels D. The Measurement of Multiple Chronic Diseases—A Systematic Review on Existing Multimorbidity Indices J Gerontol A Biol Sci Med Sci 2011;66(3):301-11.

2.  [http://www.hscic.gov.uk/qofbrv30](https://mail.campus.gla.ac.uk/owa/redir.aspx?SURL=BoFxhmAieTy1BHVVW7pCMiKcq4LYfQI0ZKVzbG2jC6YgjTyYqYPSCGgAdAB0AHAAOgAvAC8AdwB3AHcALgBoAHMAYwBpAGMALgBnAG8AdgAuAHUAawAvAHEAbwBmAGIAcgB2ADMAMAA.&URL=http%3a%2f%2fwww.hscic.gov.uk%2fqofbrv30)

3. I[http://www.isdscotland.org/Health-Topics/General-Practice/GP-Consultations/Grouping-clinical-codes.asp](https://mail.campus.gla.ac.uk/owa/redir.aspx?SURL=hHtoJjAFsgbQOd4jHw-c1S477gUsZDuX7i0-WE-tbWggjTyYqYPSCGgAdAB0AHAAOgAvAC8AdwB3AHcALgBpAHMAZABzAGMAbwB0AGwAYQBuAGQALgBvAHIAZwAvAEgAZQBhAGwAdABoAC0AVABvAHAAaQBjAHMALwBHAGUAbgBlAHIAYQBsAC0AUAByAGEAYwB0AGkAYwBlAC8ARwBQAC0AQwBvAG4AcwB1AGwAdABhAHQAaQBvAG4AcwAvAEcAcgBvAHUAcABpAG4AZwAtAGMAbABpAG4AaQBjAGEAbAAtAGMAbwBkAGUAcwAuAGEAcwBwAA..&URL=http%3a%2f%2fwww.isdscotland.org%2fHealth-Topics%2fGeneral-Practice%2fGP-Consultations%2fGrouping-clinical-codes.asp), 2008.

4. Eachus J, Williams M, Chan P, et al. Deprivation and cause specific morbidity: evidence from the Somerset and Avon survey of health. BMJ 1996;312(7026):287-92.

### Appendix 2. Read Codes used to define the presence of intellectual disability

Eu814 moderate learning disability

Eu815 severe learning disability

Eu816 mild learning disability

Eu817 profound learning disability

918e. On learning disability register

9HB3. Learning disabilities health assessment

9HB5. Learning disabilities annual health assessment

E3... Mental retardation

E30.. Mild mental retardation, IQ in range 50-70

E31.. Other specified mental retardation

E310. Moderate mental retardation, IQ in range 35-49

E311. Severe mental retardation, IQ in range 20-34

E312. Profound mental retardation with IQ less than 20

E31z. Other specified mental retardation NOS

E3y.. Other specified mental retardation

E3z.. Mental retardation NOS

Eu7.. [X]Mental retardation

Eu70. [X]Mild mental retardation

Eu700 [X]Mld mental retard with statement no or min impairm behav

Eu701 [X]Mld mental retard sig impairment behav req attent/treatmt

Eu70y [X]Mild mental retardation, other impairments of behaviour

Eu70z [X]Mild mental retardation without mention impairment behav

Eu71. [X]Moderate mental retardation

Eu710 [X]Mod mental retard with statement no or min impairm behav

Eu711 [X]Mod mental retard sig impairment behav req attent/treatmt

Eu71y [X]Mod retard oth behav impair

Eu71z [X]Mod mental retardation without mention impairment behav

Eu72. [X]Severe mental retardation

Eu720 [X]Sev mental retard with statement no or min impairm behav

Eu721 [X]Sev mental retard sig impairment behav req attent/treatmt

Eu72y [X]Severe mental retardation, other impairments of behaviour

Eu72z [X]Sev mental retardation without mention impairment behav

Eu73. [X]Profound mental retardation

Eu730 [X]Profound ment retrd wth statement no or min impairm behav

Eu731 [X]Profound ment retard sig impairmnt behav req attent/treat

Eu73y [X]Profound mental retardation, other impairments of behavr

Eu73z [X]Prfnd mental retardation without mention impairment behav

Eu7y. [X]Other mental retardation

Eu7y0 [X]Oth mental retard with statement no or min impairm behav

Eu7y1 [X]Oth mental retard sig impairment behav req attent/treatmt

Eu7yy [X]Other mental retardation, other impairments of behaviour

Eu7yz [X]Other mental retardation without mention impairment behav

Eu7z. [X]Unspecified mental retardation

Eu7z0 [X]Unsp mental retard with statement no or min impairm behav

Eu7z1 [X]Unsp mentl retard sig impairment behav req attent/treatmt

Eu7zy [X]Unspecified mental retardatn, other impairments of behav

Eu7zz [X]Unsp mental retardation without mention impairment behav
